# Knowledge mapping of graph neural networks for drug discovery: a bibliometric and visualized analysis

**DOI:** 10.3389/fphar.2024.1393415

**Published:** 2024-05-10

**Authors:** Rufan Yao, Zhenhua Shen, Xinyi Xu, Guixia Ling, Rongwu Xiang, Tingyan Song, Fei Zhai, Yuxuan Zhai

**Affiliations:** Faculty of Medical Device, Shenyang Pharmaceutical University, Shenyang, China

**Keywords:** bibliometric analysis, graph neural network, drug discovery, VOSviewer, Citespace

## Abstract

**Introduction:**

In recent years, graph neural network has been extensively applied to drug discovery research. Although researchers have made significant progress in this field, there is less research on bibliometrics. The purpose of this study is to conduct a comprehensive bibliometric analysis of graph neural network applications in drug discovery in order to identify current research hotspots and trends, as well as serve as a reference for future research.

**Methods:**

Publications from 2017 to 2023 about the application of graph neural network in drug discovery were collected from the Web of Science Core Collection. Bibliometrix, VOSviewer, and Citespace were mainly used for bibliometric studies.

**Results and Discussion:**

In this paper, a total of 652 papers from 48 countries/regions were included. Research interest in this field is continuously increasing. China and the United States have a significant advantage in terms of funding, the number of publications, and collaborations with other institutions and countries. Although some cooperation networks have been formed in this field, extensive worldwide cooperation still needs to be strengthened. The results of the keyword analysis clarified that graph neural network has primarily been applied to drug-target interaction, drug repurposing, and drug-drug interaction, while graph convolutional neural network and its related optimization methods are currently the core algorithms in this field. Data availability and ethical supervision, balancing computing resources, and developing novel graph neural network models with better interpretability are the key technical issues currently faced. This paper analyzes the current state, hot spots, and trends of graph neural network applications in drug discovery through bibliometric approaches, as well as the current issues and challenges in this field. These findings provide researchers with valuable insights on the current status and future directions of this field.

## 1 Introduction

Drug discovery is the first stage in the process of drug development, which is both costly and time-consuming. It entails testing and experimenting with thousands of compounds to identify safe and effective drugs (Schneider et al., 2020). In order to tackle this difficulty, researchers have begun to experiment with novel methods to save time and financial costs, and applying artificial intelligence to the field of drug discovery is one of them (Paul et al., 2020). Artificial Intelligence (AI) is a technology that focuses on the application of computer programs to simulate human intelligent behavior. It involves several fields such as informatics, mathematics, and biology (Shen et al., 2022a). Artificial neural networks, a fundamental technology in the field of AI, have received growing interest in recent years. For example, algorithms such as convolutional neural networks (CNN), recurrent neural networks (RNN), and autoencoders can automatically capture useful feature information. This addresses the previous requirement where traditional machine learning algorithms had to depend on the manual extraction of information features (Yazdani-Jahromi et al., 2022). However, traditional algorithms can only handle Euclidean spatial data but have limitations in processing non-Euclidean spatial data, such as social networks and biological networks. Therefore, researchers have used the concept of deep learning models, such as CNN and RNN, to establish and develop a novel artificial neural network called the graph neural network (GNN) for processing graph data (Wu et al., 2021a). GNN has shown excellent performance in processing non-Euclidean spatial data and has been widely used for traffic prediction (Cui et al., 2020; Zhao et al., 2020), recommendation systems (Zhang and Yang, 2019; Wu et al., 2022), and other fields.

Drug discovery also involves a large number of molecular structures and relationships between compounds, which may be represented as graph data. For example, in the molecular structure data of drugs, the atomic species may be regarded as the nodes of the graph, and the chemical bond types may be seen as the edges of the graph (Liu et al., 2023a; Ma and Lei, 2023). These graph data may be used to characterize the topology of molecules, chemical features, and other important information to screen and design new drug candidates. Aside from the molecular structure of the drug, several networks that exhibit interaction relationships may also be regarded as graph data. These networks include the interaction network between drugs, the interaction network between a drug and a target, and the interaction network between proteins (Zhao et al., 2021; Shao et al., 2022; Liu et al., 2023b). Therefore, researchers began to apply GNN in the field of drug development, with the aim of using graph data to improve and optimize the process of drug discovery. In 2016, Kearnes proposed (Kearnes et al., 2016) applying graph convolutional networks (GCN) to extract features from molecular graphs, which allowed the model to better utilize the information contained within the graph structure. In the following year, Pande combined (Altae-Tran et al., 2017) GCN with iterative refinement long-short-term memory networks, which significantly improved the learning of meaningful distance metrics over small molecules. These advancements marked the beginning of the application of GNN in the field of drug discovery.

As GNN technology is increasingly applied to the drug discovery field, it has demonstrated outstanding performance in various aspects. In contrast to traditional machine learning algorithms, GNN has the capability to directly analyze the graph structure of a molecule or protein, which naturally expresses the atomic structure inside the molecule and the interactions between them. Simultaneously, GNN automatically learns the representation of molecules through graph embedding and integrates multi-modal data, which has obvious advantages in understanding the multilevel mechanism of drug action and improving prediction accuracy (Xiong et al., 2020; Zhou et al., 2020). GNN can be trained to predict multiple target tasks at the same time, such as predicting the solubility and toxicity of molecules at the same time. This approach eliminates the need for traditional machine learning algorithms to create separate models for each prediction task and overcomes the challenge of enabling knowledge sharing among multiple independent models (Stokes et al., 2020). While GNN has obvious advantages in drug discovery, it also presents challenges such as the insufficient interpretability of the model, the need for large amounts of labeled data for training, and the high consumption of computing resources (Zhou et al., 2020; Wu et al., 2021a). Therefore, scientists are constantly exploring and improving GNN, and the output of related research results has been increasing. However, it is difficult for researchers to grasp the latest progress and research hotspots in the field from numerous research results. Hence, summarizing the development status and research hotspots is crucial for establishing research directions and guiding future research. Bibliometric analysis is an information visualization tool that offers researchers who have been or will be engaged in the field a scientific and reliable analysis of the research dynamics. For example, scholars can analyze the present status of research across different nations, institutions, authors, and publications to discover active researchers, investigate new collaboration opportunities, and examine the present research hotspot and trend by examining the highly cited papers, reference burst detection, keyword co-occurrence network, and thematic map.

This study aims to provide a comprehensive overview of GNN in the field of drug discovery over the last 7 years, utilizing bibliometric analysis and discussing the following aspects:

The pace of development of GNN applications in drug discovery from 2017 to 2023.

The distribution and cooperation status of main countries, authors, institutions, and journals in the field of GNN applications in drug discovery.

The research hot spots and emerging developments in the field of GNN applications in drug discovery.

The paper is organized into four distinct sections: The first part introduces the background of the article and the bibliometric methodology. The second part describes the approach used for collecting and processing data. The third part presents various aspects of the collected publications, including the quantity across different years, countries, institutions, journals, and authors, as well as papers frequently cited in the field and frequently occurring keywords. The fourth part summarizes and discusses these aspects, focusing on the research hot spots, trends, and unresolved issues of GNN applications in drug discovery.

## 2 Material and methods

### 2.1 Data sources

We used the Science Citation Index Expanded (SCI-Expanded 2002–present) from Clarivate Analytics’ Web of Science Core Collection (WoSCC) as our data source. WoSCC is a professional and authoritative citation database with a powerful indexing function that is widely used in bibliometric research.

### 2.2 Data retrieval strategy

**FIGURE 1:**
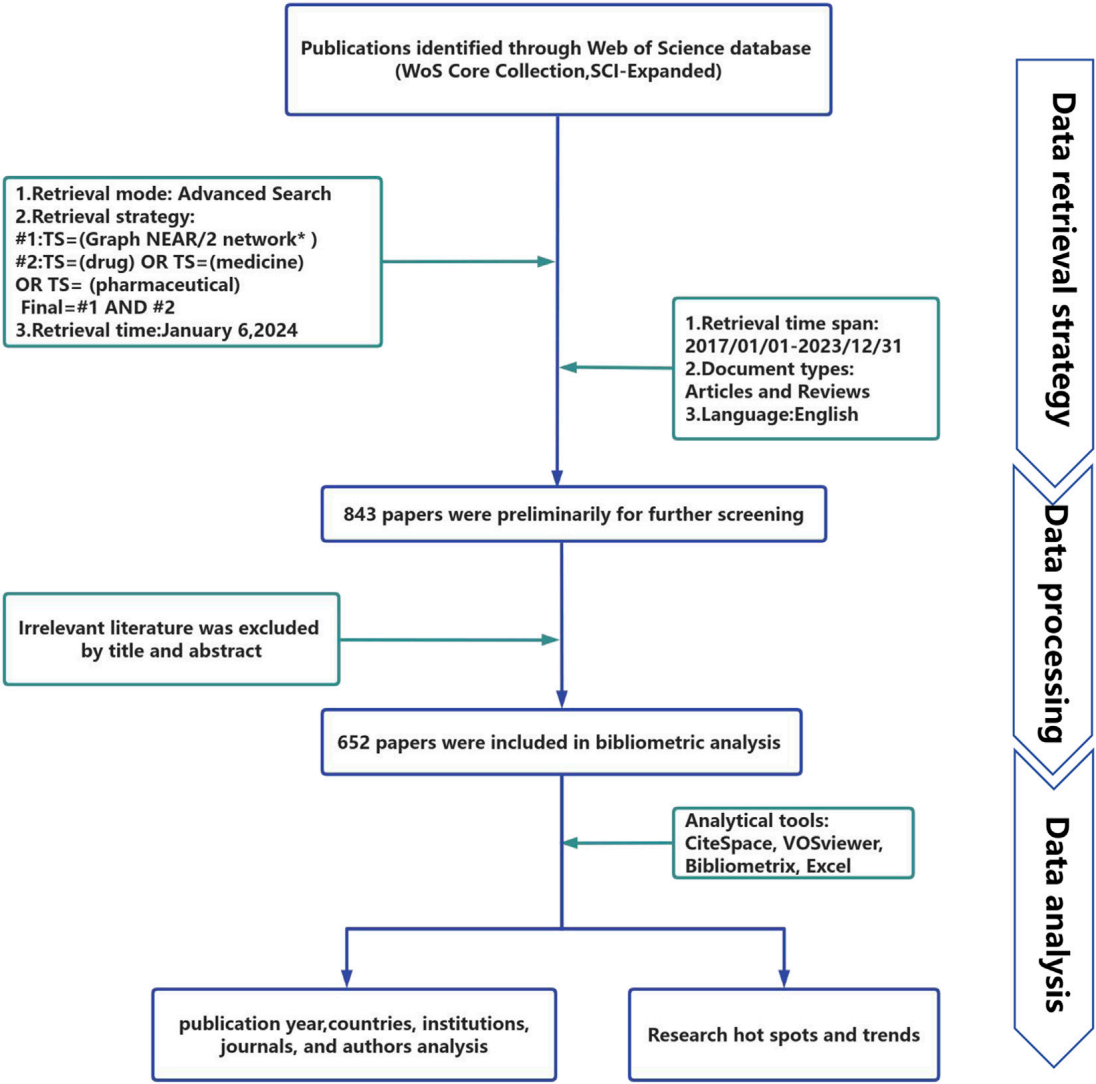
Flowchart of the publication search and selection process.

In this study, all of the obtained publications were retrieved and downloaded from the WoSCC database on 6 January 2024, using the following search equation for publication collection: #1: TS = (graph NEAR/2 network*); #2: TS = (drug) OR TS = (medicine) OR TS = (pharmaceutical); and the final search equation was #1 AND #2. To obtain as many relevant sources as possible, wildcard characters (*) were used to represent one or more other characters and allow for variable endings of keywords. For example, network* also includes the plural of a network, networks. The use of NEAR/2 specifies that the maximum number of words separating search terms connected by this operator is 2. Examples included topics such as graph neural networks and graph neural convolutional networks. The literature publication period was 2017–2023, and the language was limited to English. The publication type was limited to articles and reviews. Figure 1 presents the specific exclusion criteria.

### 2.3 Data processing

We retrieved a total of 843 literature candidates. The titles and abstracts of the literature were then manually checked to exclude literature unrelated to the research topic. Finally, 652 pieces of literature were included for the data analysis. Table 1 summarizes the relevant information from these papers. All 652 retrieved papers were downloaded as “Full Record and Cited References” and exported in plain text format. Some of the inherent shortcomings of the WOS database were checked and merged, and information from various regions was included in their affiliated countries. For example, publications from England, Northern Ireland, Scotland, and Wales were assigned to the UK (Cheng et al., 2022).

**TABLE 1:** Main information about data.

Description	Results
Timespan	2017:2023
Sources (Journals, Books, etc.)	168
Papers	652
article	619
article; data paper	4
article; proceedings paper	13
review	16
Annual Growth Rate %	149.8
Paper Average Age	2.04
Average citations per doc	13.29
References	19,174
Keywords Plus	836
Author’s Keywords	1215
Authors	2654
Authors of single-authored docs	2

### 2.4 Analytical tools

Microsoft Excel 2019 and the R package bibliometrix (Aria and Cuccurullo, 2017) were used to perform the basic statistical analyses of the annual number of publications and citations, countries/regions, institutions, authors, funding agencies, journals, and keywords. The country/institution/author collaboration analysis, the author/journal co-citation analysis, and the keyword co-occurrence analysis were performed using VOSviewer 1.6.19. The journal discipline distribution and reference burst detection were performed using CiteSpace V6.1. Bibliometrix is a new bibliometrics software introduced in 2017 that can perform comprehensive science mapping analysis. It is an open-source tool for programming with R that can be quickly upgraded and integrated with other statistical R packages. CiteSpace, developed by Prof. Chaomei Chen, is another tool for visualizing and constructing bibliometric networks by creating visual maps of specific literature to analyze the current state of research and infer trends (Chen, 2006; Chen et al., 2014).

VOSviewer is a free Java-based soft bibliometric analysis piece that provides three types of visualization maps, including network visualization, overlay visualization, and density visualization (van Eck and Waltman, 2010). Generally, in these visualization maps, different nodes represent different items such as authors, countries, institutions, journals, and keywords, and the size of the nodes reflects the number of published papers, publication citations, or occurrence frequency for the corresponding items. Links between nodes represent co-citations or co-occurrence associations between nodes. However, the layout of the cooperative network diagram is crucial to visualizing and understanding the results. In this study, by adjusting the hyperparameters of VOSviewer, Attraction and Repulsion are set to 3 and −3, respectively, to obtain the best network layout. Such a setting can make the nodes of the network map better distributed in space, reduce overlap and congestion, so that the cooperative relationship is more clearly displayed, and help researchers better understand the cooperative relationship between the literature.

## 3 Results

### 3.1 Publication and citation trends

**FIGURE 2:**
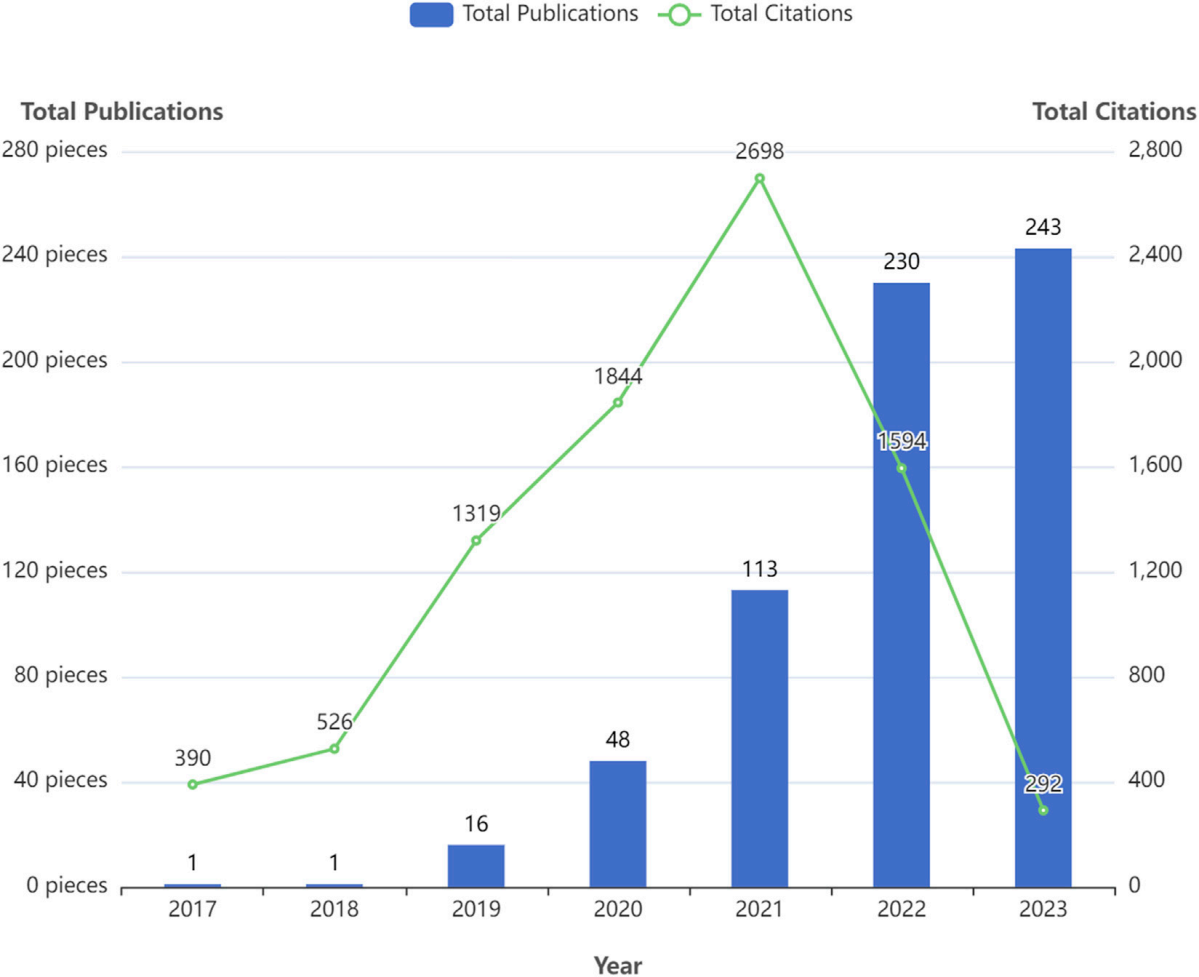
Overall trend in the number of publications over the past 7 years.

Figure 2 displays the annual number of GNN publications in the field of drug research. There was only one relevant paper published in 2017 and 2018. Since 2019, the number of papers published has been increasing, reaching 243 in 2023. In the first 5 years, the citation frequency of papers increased continuously. The slight decrease in the last 2 years is also related to the recent proximity of the current analysis dates. Table 2 shows the annual paper count and citation frequency. TP represents total publications, and TC represents total citations. And four thresholds for the number of paper citations are described: the number of papers with equal to or more than 100, 10, 1, and 0 citations. To a certain extent, these four thresholds for the number of citations in papers reflect the paper’s quality.

**TABLE 2:** Annual scientific production and average citations per year.

Year	TP	TC	≥10^2^	≥10^1^	≥10^0^	0	TC/TP
2017	1	390	1	1	1	0	390
2018	1	526	1	1	1	0	526
2019	16	1319	4	16	16	0	82.44
2020	48	1844	5	39	48	0	38.42
2021	113	2698	4	67	110	3	23.88
2022	230	1594	0	57	199	31	6.93
2023	243	292	0	4	110	133	1.2
Total	652	8663	15	185	485	167	
Percentage	100%	_	2.30%	28.37%	74.39%	25.61%	

**Note:** TP: total publications, TC: total citations; ≥10^2^, ≥10^1^, ≥10^0^, 0: Number of papers with equal or more 100,10, 1 and 0 citations.

### 3.2 Country/region analysis

**FIGURE 3:**
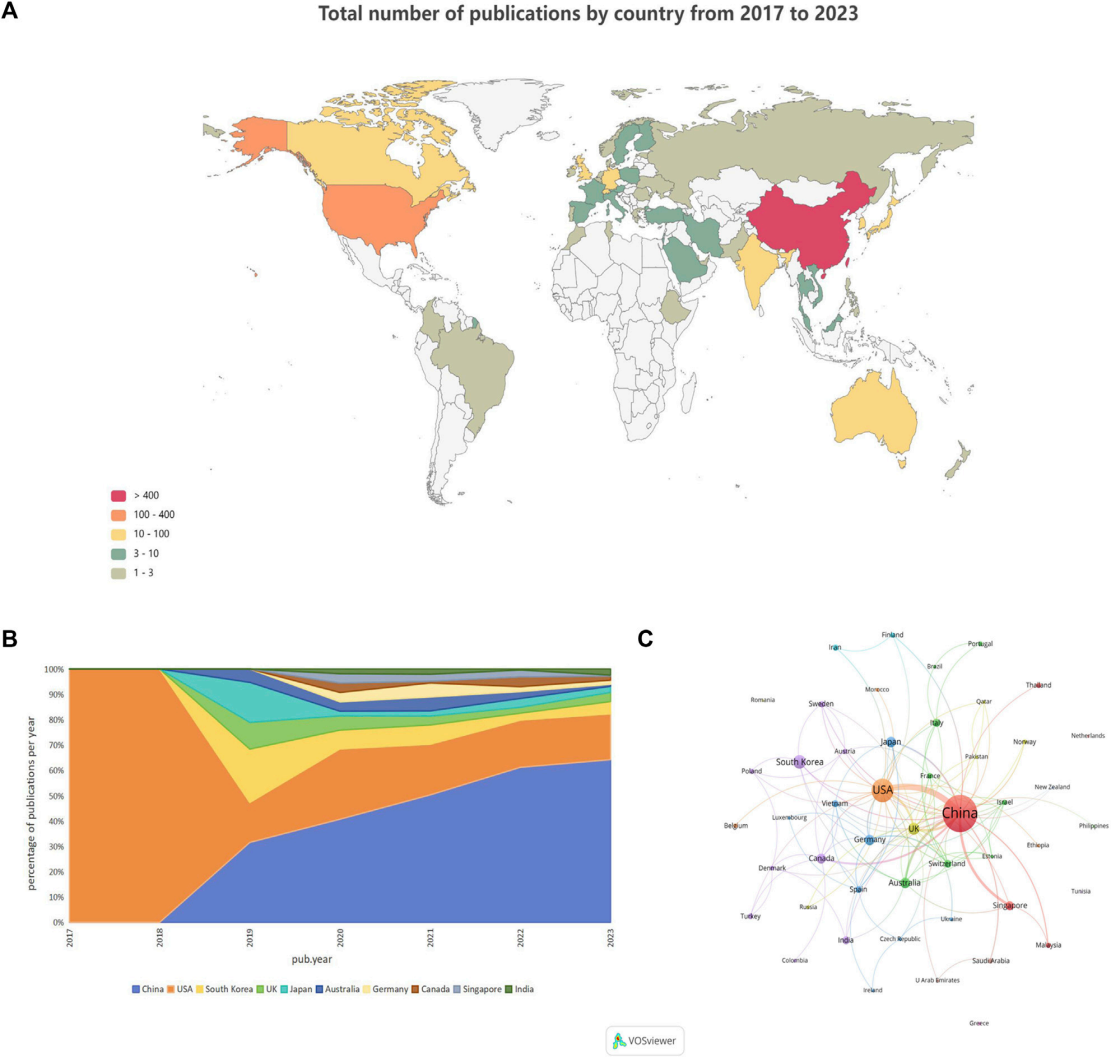
Country/region analysis **(A)**. Geographical distribution of the total number of publications in the different countries and regions **(B)**. Percentage of annual publications in the top 10 countries over the past 7 years **(C)**. Visualization of country cooperation.

Among the 652 papers included, a total of 48 countries/regions were found to be involved in the research work in this field. Figure 3A shows the geographical distribution of the number of publications in the different countries/regions, and the countries with the most research in this field were located in Asia and North America, with Europe and Australia also involved. This result shows that the combination of GNN and drug discovery, as a newly explored research area in recent years, has not yet been studied and discussed globally. Table 3 lists the top 10 countries and regions in terms of the number of publications in this field. China, the United States, and Korea were the countries with the highest number of total publications, respectively. Figure 3B shows a plot of the top 10 countries in terms of the number of annual publications in 2017–2023. The proportion of each country in the chart represents its number of publications relative to the total number of publications in the top ten. It can be observed that only the United States published papers in this field in 2017 and 2018. However, as time progressed, other countries also began to conduct research in this field, with China’s publication proportion increasing annually. Figure 3C shows the cooperation network among the 48 countries/regions involved in issuing papers. The size of the node indicates the number of publications, and the thickness of the lines indicates the strength of cooperation. It is worth noting that China and the United States have the highest number of publications and show a close cooperative relationship.

**TABLE 3:** Top 10 highly producing countries and regions.

Rank	Country	TP	Percentage (%)	TC	TC/NP	PY_start
1	China	426	65.34	4148	9.74	2019
2	United States	142	21.78	3401	23.95	2017
3	South Korea	38	5.83	574	15.11	2019
4	United Kingdom	28	4.29	522	18.64	2019
5	Japan	21	3.22	376	17.90	2019
6	Australia	20	3.07	415	20.75	2019
7	Germany	19	2.91	238	12.53	2020
8	Canada	17	2.61	152	8.94	2020
9	Singapore	14	2.15	270	19.29	2020
10	India	11	1.69	80	7.27	2021
10	Switzerland	11	1.69	202	18.36	2021

**Note:** PY_start: Publication Year start.

### 3.3 Institutional analysis

**FIGURE 4:**
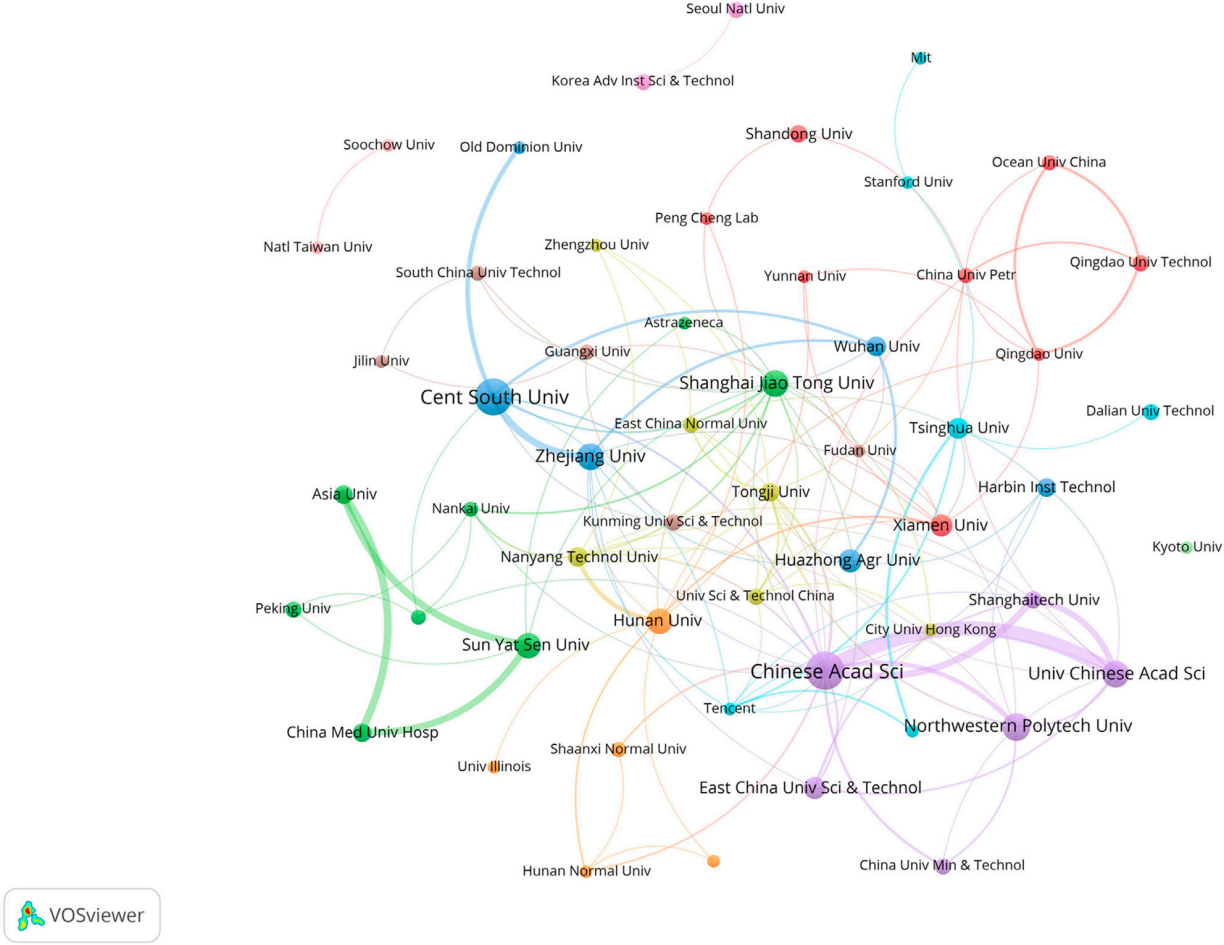
Visualization of institutional cooperation.

In this field, research institutions can reflect the distribution of scientific research units. According to a VOSviewer analysis of institutional partnerships, more than 800 institutions were involved in paper publication. The number of issuing institutions with more than or equal to five papers in the past 7 years was counted, for a total of 55 institutions. Table 4 reveals that the top 10 institutions in terms of the number of papers issued were all from China. Figure 4 illustrates the network of the institutional cooperation analysis, which VOSviewer clustered based on the cooperation relationships between these 55 institutions. The various clusters highlight the differences in the cooperation relationships and research directions among these institutions. Among them, the Chinese Academy of Science node is the largest, indicating that this institution published the most papers and had the same color as the nodes of the University Chinese Academy of Science and Northwestern Polytech University, indicating that they belonged to the same cluster (cooperation group), which suggests that the research direction of these institutions was similar and the frequency of their cooperation was also higher. In addition to cooperation and communication with domestic institutions, the Chinese Academy of Science has also established good cooperation and communication with foreign institutions, such as Nanyang Technological University.

**TABLE 4:** Major research institutions and number of publications.

Rank	Institute	TP	TC	TC/TP	Country
1	Chinese Academy Of Sciences	39	844	21.64	China
2	Central South University	36	438	12.17	China
3	Northwestern Polytechnical University	21	404	19.24	China
4	Shanghai Jiao Tong University	20	132	6.60	China
5	University Of Chinese Academy Of Sciences	19	477	25.11	China
6	Zhejiang University	19	260	13.68	China
7	Sun Yat Sen University	18	256	14.22	China
8	Hunan University	17	244	14.35	China
9	Huazhong Agricultural University	15	199	13.27	China
10	East China University Of Science & Technology	13	86	6.62	China
10	Xiamen University	13	128	9.85	China

### 3.4 Journal analysis

**FIGURE 5:**
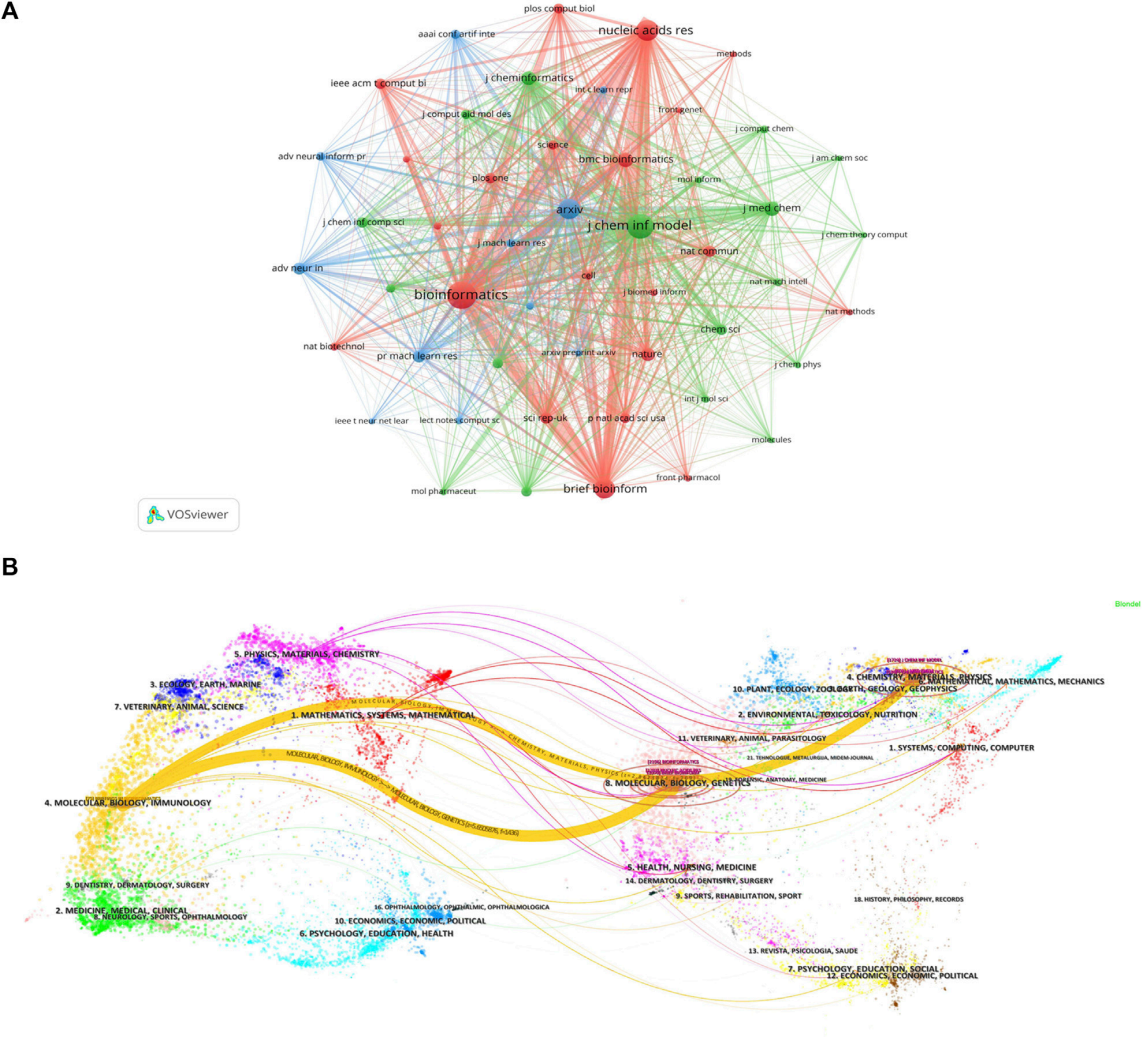
Journal analysis **(A)**. Visualization of journal co-citation analysis **(B)**. Double-graph overlay of journals.

Currently, research papers related to the application of GNN to drug discovery have been published in 168 academic journals. According to the results in Table 5, the journal Briefings in Bioinformatics had the highest number of publications with a total of 1303 citations, followed by Bioinformatics and the Journal of Chemical Information And Modeling. The total number of citations for Bioinformatics was 1580, which was higher than any other journal. According to the Journal Citation Report (JCR) 2023, the top 10 journals were located in Q1/Q2, with Briefings in Bioinformatics (IF = 9.5) having the highest impact factor (IF). Journal co-citations are also an important indicator of journal impact. In this study, 50 journals were co-cited at least 100 times, and we used VOSviewer to generate a map of journal co-citation networks (Figure 5A). The top three journals with the highest citations were Bioinformatics, Journal of Chemical Information And Modeling, and Nucleic Acids Research. Figure 5B is a double-graph overlay of journals that shows the flow of knowledge between different disciplines in this field. The graph’s left side indicates the basic disciplines of the citing journal, while the right side indicates the basic disciplines of the cited journal. The thickness of the lines shows the frequency with which the left side referenced the right side.

**TABLE 5:** Top 10 journals in terms of publications.

Rank	Journal title	h_index	TC	TP	PY_start	IF (2023)	JCR
1	Briefings In Bioinformatics	20	1303	75	2020	9.5	Q1
2	Bioinformatics	15	1580	44	2018	5.8	Q1
3	Journal of Chemical Information And Modeling	16	996	38	2019	5.6	Q1
4	Ieee-Acm Transactions on Computational Biology And Bioinformatics	8	200	31	2021	4.5	Q1\Q2
5	Bmc Bioinformatics	6	168	30	2020	3	Q2
6	International Journal of Molecular Sciences	6	170	17	2019	5.6	Q1\Q2
7	Journal of Cheminformatics	6	351	17	2019	8.6	Q1
8	Computers In Biology And Medicine	4	58	16	2022	7.7	Q1
9	Methods	5	253	14	2019	4.8	Q2
10	Frontiers In Pharmacology	4	30	12	2021	5.6	Q1
10	Ieee Access	4	50	12	2020	3.9	Q2
10	Acs Omega	3	104	12	2020	4.1	Q2

**Note:** h-index: Measured in terms of the ‘h’ number of publications with at least “h” citations.

### 3.5 Author analysis

**FIGURE 6:**
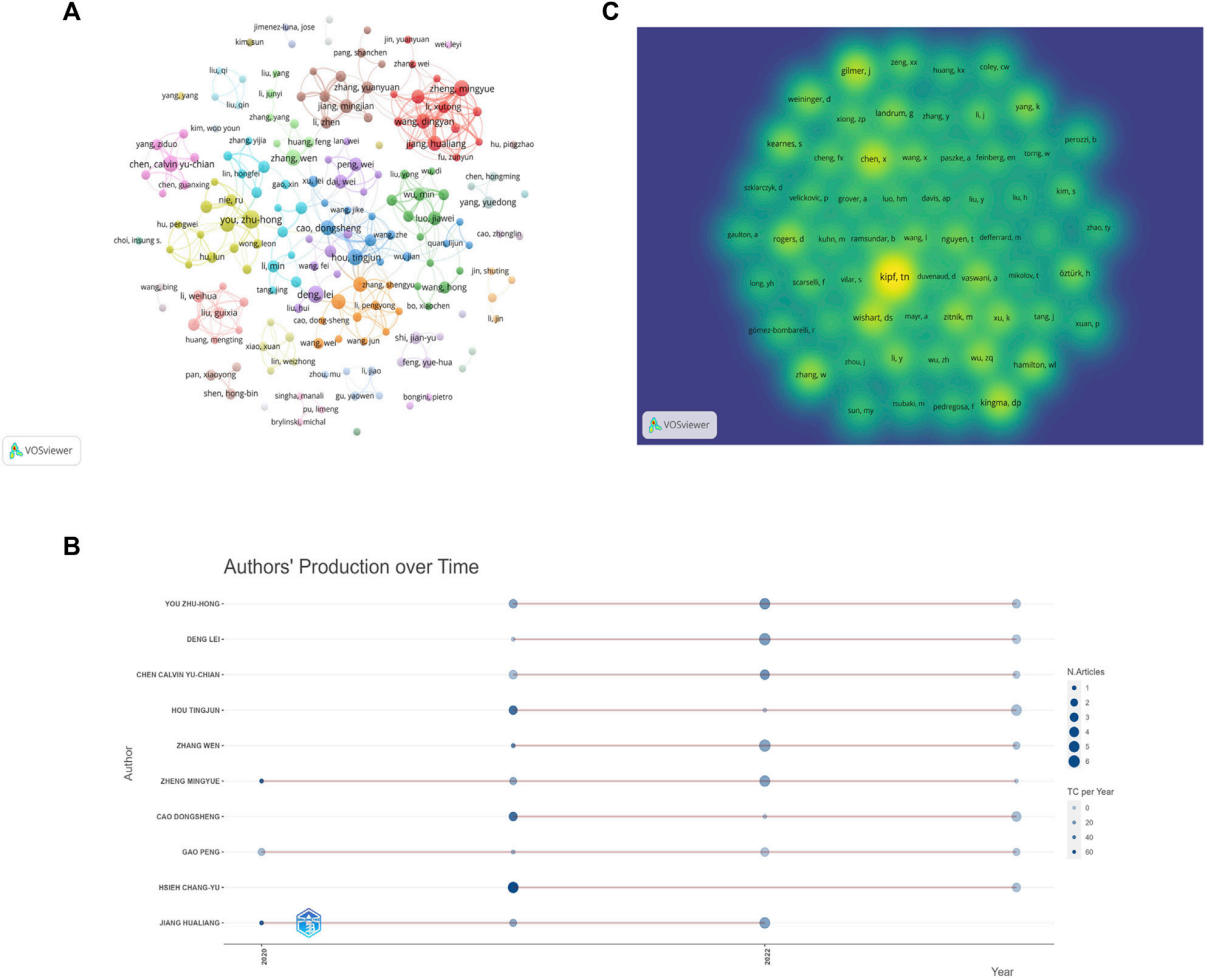
Author analysis **(A)**. Visualization of author cooperation **(B)**. Author co-citation collaboration network density map **(C)**. Top 10 Authors’ Production over Time.

The 652 papers included a total of 2654 authors. Among them, the number of papers published by the most published authors was 11. According to the Price’s law, the number of published papers should be more than 2.48 in order to be recognized as the core author. Therefore, authors with at least three papers are considered core authors. Figure 6A shows the core author collaboration analysis graph generated using the VOSviewer. The different colors represent different clusters, the size of nodes represents the number of publications, and the thickness of the lines represents the frequency of author collaboration. There were 177 authors with more than three publications, and this generated 36 clusters, indicating that there were many author cooperation groups for GNN in the field of drug discovery. Table 6 summarizes the top 10 most published authors, all of whom are from China, with You Zhu-Hong, Deng Lei, and Chen Calvin Yu-Chian ranking in the top three with eleven, ten, and nine papers, respectively. Figure 6B shows the top 10 most productive authors’ production over time; the size and color of node represent the annual number of papers and the total citations per year, respectively. Figure 6C shows the density map of the author co-citation collaboration networks, which includes 61 authors with at least 50 citations. Each point in the item density visualization has a color that indicates the density of items at that point, colors range from blue to green to yellow. The larger the number of items in the neighborhood of a point and the higher the weights of the neighboring items, the closer the color of the point is to yellow. Kipf, TN, was the only author with more than 200 citations and had the highest impact.

**TABLE 6:** Top 10 most productive authors.

Authors	Country	h_index	TC	NP	TC/NP	PY_start
You Zhu-Hong	China	7	157	11	14.27	2021
Deng Lei	China	5	74	10	7.4	2021
Zheng Mingyue	China	6	391	9	43.44	2020
Chen Calvin Yu-Chian	China	5	102	9	11.33	2021
Zhang Wen	China	5	198	9	22	2021
Hou Tingjun	China	4	199	9	22.11	2021
Jiang Hualiang	China	6	391	8	48.88	2020
Hsieh Chang-Yu	China	5	266	8	33.25	2021
Wang Dingyan	China	5	337	8	42.13	2020
Cao Dongsheng	China	4	199	8	24.88	2021

**Note:** PY_start: Publication Year start.

### 3.6 Documents analysis

**FIGURE 7:**
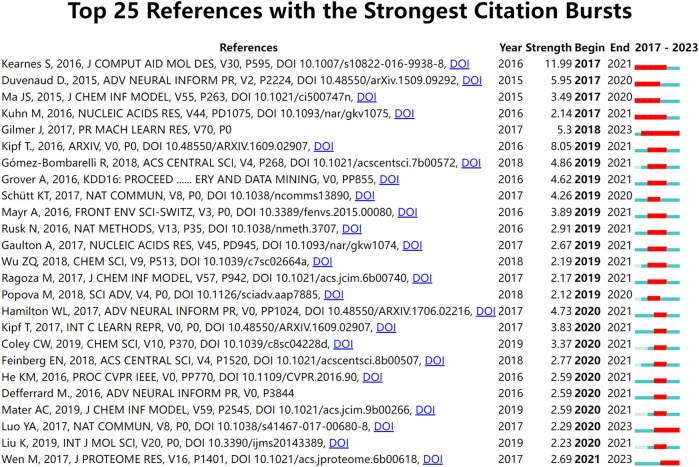
Top 25 references with the strongest citation bursts.

Papers with a high citation frequency are often representative of the field and have a great impact on its development. 15 out of 652 papers were cited more than 100 times. Table 7 shows the filtering of the highly cited TOP10 papers based on the total citation frequency. Research papers published in Bioinformatics in 2018 had the highest citation frequency, with over 500 citations (Zitnik et al., 2018), followed by paper published in ACS in 2017. the 2020 paper from the Journal of Medicinal Chemistry ranked third (Xiong et al., 2020). In addition, references with strong citation bursts were explored using CiteSpace, and Figure 7 displays the top 25 references with the strongest citation bursts, sorted by the burst’s beginning year. These strong burst of literatures provide a solid technical foundation for the application of GNN in drug discovery, which mainly include research on GNN algorithms and drug-target interaction (DTI) research.

**TABLE 7:** Top 10 highly cited papers.

Rank	Document (Author/Year/Journal)	Title	Year	Global citations	Local citations
1	Zitnik M, 2018, Bioinformatics	modeling polypharmacy side effects with graph convolutional	2018	526	81
2	Altae-Tran H, 2017, Acs Central Sci	low data drug discovery with one-shot learning	2017	390	31
3	Xiong Zp, 2020, J Med Chem	pushing the boundaries of molecular representation for drug discovery with the graph attention mechanism	2020	273	72
4	Tsubaki M, 2019, Bioinformatics	compound-protein interaction prediction with end-to-end learning of neural networks for graphs and sequences	2019	260	53
5	Nguyen T, 2021, Bioinformatics	graphdta: predicting drug-target binding affinity with graph neural networks	2021	215	61
6	Sun My, 2020, Brief Bioinform	graph convolutional networks for computational drug development and discovery	2020	182	48
7	Li Y, 2019, Methods	deep learning in bioinformatics: introduction, application, and perspective in the big data era	2019	168	1
8	Lim J, 2019, J Chem Inf Model	predicting drug-target interaction using a novel graph neural network with 3days structure-embedded graph representation	2019	166	35
9	Jiang Dj, 2021, J Cheminformatics	could graph neural networks learn better molecular representation for drug discovery? a comparison study of descriptor-based and graph-based modelsSource	2021	166	26
10	Torng W, 2019, J Chem Inf Model	graph convolutional neural networks for predicting drug-target interactions	2019	153	42

### 3.7 Keyword analysis

**FIGURE 8:**
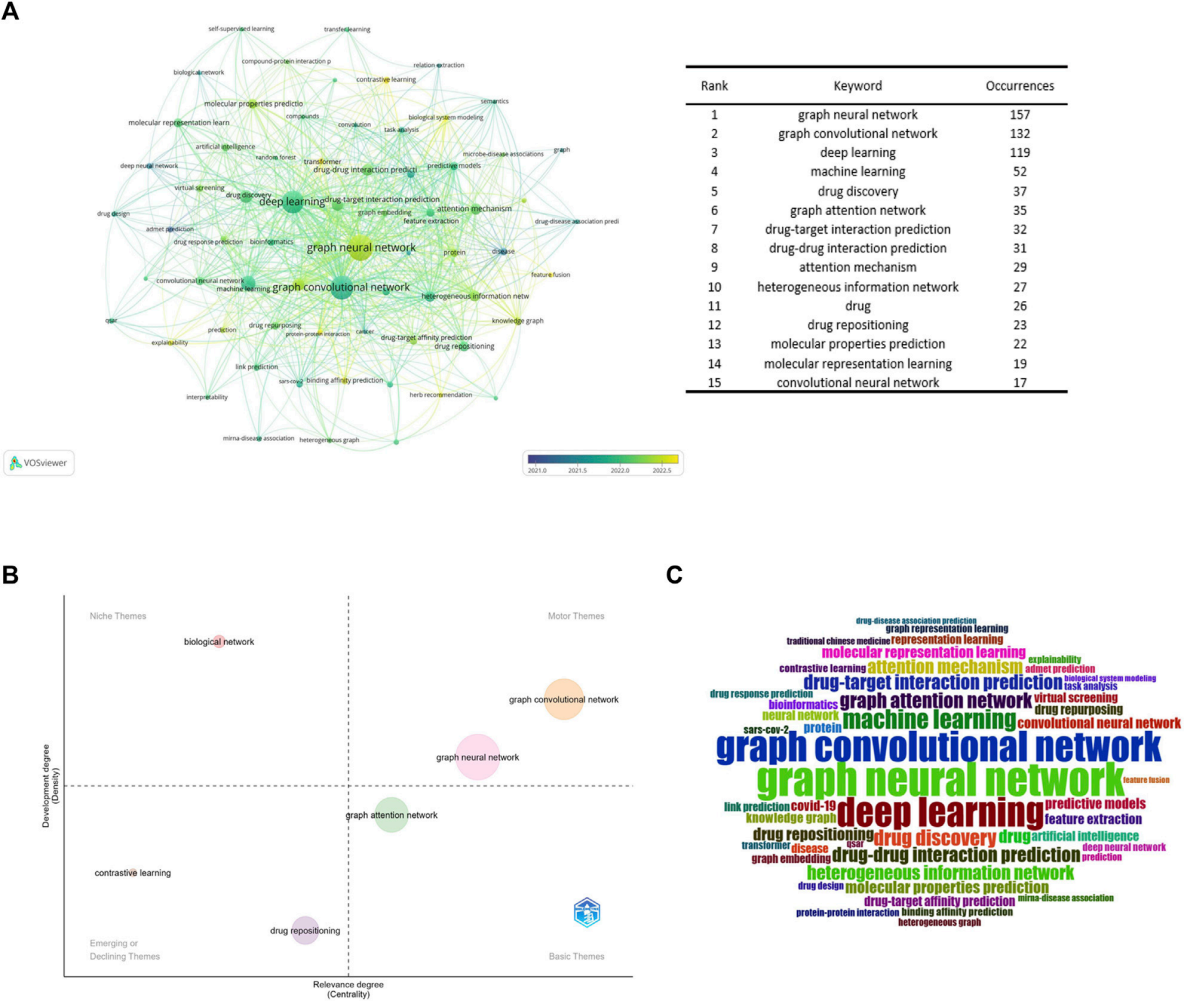
Keyword analysis **(A)**. Keyword collaboration network overlay visualization map **(B)**. Strategic diagram representing research trends of GNN in drug discovery **(C)**. Keywords’ wordcolud.

The VOSviewer program was used to build a high-frequency keyword co-occurrence network map. By assessing the frequency of keyword occurrences, research hotspots and developing research trends in this sector were identified. Figure 8A displays the high-frequency keyword co-occurrence maps produced. The larger the nodes in the graph, the higher the co-occurrence frequency of the keywords they represent, and the closer the color of the nodes is to yellow, which indicates that the keywords appeared later. Figure 8B is a thematic map of keywords created with Bibliometrix (Cobo et al., 2011; Ampah et al., 2021), which uses the walk trap clustering algorithm with each cluster displaying only one label. The quadrants represent different thematic spaces: central and developed, peripheral and developed, peripheral and undeveloped (emerging or declining themes), and central and undeveloped, reflecting the emergence of major themes and new themes in the field since 2017. Figure 8C is a word cloud of keywords.

## 4 Discussions

Bibliometrics is an interdisciplinary field that uses mathematical and statistical techniques to quantify and visually examine literature. Unlike systematic reviews and meta-analyses, bibliometric visualization approaches, such as VOSviewer and CiteSpace, provide a more intuitive analysis of the current status, development trends, and hotspots of a research field (Merigo and Yang, 2017). The assessment findings show enhanced reliability and repeatability. This study provides a comprehensive overview of the current state of GNN applications in the field of drug discovery. We conducted a bibliometric analysis to summarize the existing research and presented the results visually using different software tools. The analysis includes insights into the development trends and future research hotspots in this field.

### 4.1 Discussions of publication year, leading countries, institutions, journals, and authors

To a certain extent, the number of scientific papers published reflects the development of research in a particular field (Shen et al., 2022b). The changes in the number of publications during different periods reflect the theoretical level and speed of the field’s academic research development. According to the results of the annual distribution of publications, only one relevant paper was published in 2017 and 2018, both from Stanford University in the United States. Since then, the application of GNN in drug discovery has gradually entered the researchers’ minds. In 2020, there was a significant increase in the number of annual papers, and 2021 and 2022 showed explosive growth. In terms of the number of papers cited, TC/TP indicates the average number of citations per paper, which aids in evaluating the average impact and quality of published research outputs. Higher TC/TP values typically indicate a greater impact and higher quality of research outputs. The average number of citations per paper between 2017 and 2021 exceeded 25, indicating that the research results in this field have a high degree of academic impact and paper quality. In addition, 74.39% of papers were cited more than 1, and the 2023 papers’ TC/TP was also more than 1. These results demonstrate that research on GNN in the drug field is a topic of great interest to the scientific community. These were attributed to the fact that many excellent researchers have discussed the expressive ability of various GNN architectures in depth. Gradually, GNN technology has matured and gained widespread use. Therefore, the application of GNN in the drug discovery field has gradually attracted people’s attention. The field is at a high growth stage, with broad prospects and good development.

In terms of the country/region distribution results, China (426 papers, 65.34%) had the highest number of published papers, followed by the United States (142 papers, 21.78%). China and the United States accounted for 3/4 of the total number of publications in this field, indicating their leading position in this research area. However, although the number of publications in China was approximately two times that of the United States, the total citation frequency of papers was nearly the same. This is because the United States has taken the lead in this field since 2017 and has published several high-quality papers consecutively, which have become important papers for other researchers to study and refer to repeatedly. Figure 3B demonstrates that the United States initiated the field study, publishing the only paper in 2017 and 2018. China began publishing research results in this field in 2019 and has already published some classic papers in high-quality journals (Supplementary Table S1). For example, Jiang’s team’s paper on predictive molecular property modeling in the Journal of Medicinal Chemistry has been cited up to 274 times, indicating that these Chinese research teams’ output has been widely recognized and cited in the field. However, in terms of average citation frequency, the United States and Australia both have an average citation frequency of more than 20, especially the United States, which is clearly leading in terms of the number and quality of papers. China’s average citation frequency is only higher than India’s and Canada’s in the top ten. It is also clear from Supplementary Table S1 that most of China’s highly cited papers were published in 2020 and 2021, indicating that citations usually take some time to accumulate. China consistently demonstrates a strong research fervor in this field, publishing numerous papers in 2022 and 2023, thereby enhancing its academic value and influence in this field over time.

The mapping of national cooperation networks also showed that 44 of the 48 countries and regions involved in the publications have established cooperation with other countries and regions in field research. Among them, China has established international research collaborations with several countries/regions around the world and is the country with the most extensive international collaborative research in this field. For example, the United States, Australia, Singapore, Canada, and the United Kingdom. However, North America, Asia, and some European countries dominated the most collaborative research; there remained a small number of individual nodes in the network that had not established cooperative relationships with other countries. Four countries—Greece (Harigua-Souiai et al., 2021), Netherlands (Reau et al., 2023), Tunisia (Vella and Ebejer, 2022), and Romania (Albu et al., 2023)—have not yet constructed collaborative relationships with other countries, probably because these countries have initially begun to conduct research in this field and still need to strengthen international communication and cooperation. It is undeniable that financial support also plays an important role in supporting research output. When we look at the number of publications involving funding agencies (Supplementary Table S2), the top 10 funding agencies (there was a tie for 10th place) in this research area were primarily located in China (5 agencies), the United States (3 agencies), and Korea (2 agencies), with the National Natural Science Foundation of China (NSFC), the National Key Research and Development Program of China, and the National Institutes of Health (NIH USA) being the primary funders. This result clearly demonstrates the close relationship between China’s and the United States’ leading positions in this field and their robust financial support for research. This suggests that countries that value research and development in this field need to increase their financial support for scientific research so that they may become important players in this field in the future.

Among the research institutions involved in publishing papers in this research field, the Chinese Academy of Science had the most publications (39 papers), indicating that the Chinese Academy of Sciences holds a leading position in this field. According to the layout of the entire map (Figure 4), Chinese research institutions have intensive internal cooperation as well as extensive cooperation with other research institutions, thereby forming a certain scale of stable cooperation relationships in China. The Chinese Academy of Sciences had frequent contact with Shanghai Technology University and the East China University of Science and Technology (Xiong et al., 2021; Wan et al., 2022). It has also established cooperation with the Central Southern University (Wu et al., 2021b), which has a large number of publications. Most of the Chinese institutions shown in Figure 4 have only established contacts with domestic institutions, and a few of them cooperate with foreign universities and research institutes. International institutions have yet to form a sizable cooperative group. Korean institutions, such as the Korea Advanced Institute of Science and Technology and Seoul National University, have also established only internal collaborative networks (Ryu et al., 2019) without communicating with other international institutions. This may be due to the fact that applying GNN to the field of drug discovery is still a relatively new topic, and many institutions have just begun their research.

In terms of institutional distribution, the research institutions with a high number of publications were primarily located in the Asian region, with more universities in China, South Korea, and Singapore, indicating a high level of interest in the field in these countries. Research institutions consist mostly of research institutes, colleges, and universities that possess significant research capabilities, ample resources, and a high level of research excellence. In addition to higher education and research institutes, there were also pharmaceutical companies, hospitals, and other organizations. These institutions can provide university laboratories with experimental data to test models. The DeepDDS model developed by Nanjing Tech Univ, Sch Comp Sci & Technol’s team (Wang et al., 2022a) to predict the synergistic effects of drug combinations used an independent test set released by AstraZeneca to verify the predictive accuracy of the model. In addition, pharmaceutical companies and hospitals can use the prediction results provided by universities for experimental validation to determine whether they can enter clinical use. The two complement each other’s strengths and promote each other.

Journals are an important vehicle for presenting academic information and knowledge dissemination results, and journal analysis can provide researchers with a large amount of reliable reference information that helps them identify high-quality and appropriate target journals when searching the literature or submitting research. In addition to the total citation frequency, the impact factor (IF) (Garfield, 2006) and JCR category are also important indicators for evaluating the academic status of journals. The top 10 journals, in terms of the number of publications, were primarily in the fields of bioinformatics and cheminformatics. These journals were all located in Q1/Q2, with an average IF value of 5.73 and a total of 318 papers, accounting for 48.8% of the total number of publications. The finding indicates that the majority of the papers related to the application of GNN in drug discovery were published in high-impact journals. A journal co-citation analysis reflects the connection between different research results. Among these, Bioinformatics was cited more than 2,000 times, demonstrating that these journals have a higher likelihood of being cited for research papers related to GNN applications in this research field. Figure 5A reveals that Bioinformatics frequently receives citations alongside the Journal of Chemical Information Models, Brief Bioinformatics, and Nucleic Acids Research, indicating their high relevance in this field. Additionally, Bioinformatics and the Journal of Chemical Information Models ranked among the top three in terms of published papers, demonstrating their great influence, and these journals have a higher likelihood of being cited for research papers related to GNN applications.

Figure 5B is a double-graph overlay of journals. The left side represents the basic disciplines of the citing journals, also referred to as the knowledge frontier, while the right side represents the subject discipline of the cited journal, also known as the knowledge base. The double-graph superposition graph of the journal can show the reference relationship between the knowledge frontier and the knowledge foundation when the GNN is applied to drug research from a macro perspective and grasp the historical trajectory of discipline development. Figure 5B clearly displays two prominent yellow lines, symbolizing the primary citation pathways in this field. The themes on the cited side (right side), Theme 4 (#4 CHEMISTRY, MATERIALS, PHYSICS), and Theme 8 (#8 MOLECULAR, BIOLOGY, GENETICS), were cited 769 and 1436 times, respectively, by the themes on the citing side (left side), Theme 4 (#4 MOLECULAR, BIOLOGY, IMMUNOLOGY). The two subject topics converge into one subject on the cited side, and the development pattern of the subject shows a confluence state, indicating that the application of GNN in drug research requires knowledge from multiple disciplines. The field now involves molecular, biological, and immunological disciplines.

Among the core authors published in this field, the team represented by Zheng Mingyue and Jiang Hualing had close collaboration and a large network layout, both of whom belong to the Institute of the Shanghai Institute for Advanced Immunochemical Studies (SIAIS), and they had more outputs and maintained a high scientific impact during 2020–2023. The most-cited paper by the team was published in 2020. As the most published author, You Zhu-Hong had published numerous high-quality papers between 2021 and 2023, and his H-index was at a high level, with the main research direction of miRNA-disease associations prediction (Li et al., 2021). In addition, the author, Zitnik Marinka, is also a core author and has begun research in this field in 2018, and the total number of citations for the author reached 500, which demonstrates the outstanding contribution and importance of the author in this field. And although there are clusters and more collaborative groups in Figure 6A, most of them were internal collaborations with fewer communication links with other groups. Future studies should strengthen external collaboration. Table 6 shows that the top 10 authors with the most papers were all Chinese authors, indicating that China pays more attention to research in this field compared to other countries. As for the author co-citation analysis, the highest co-cited author was Kipf, TN, indicating that Kipf is a very influential author. His paper, “Semi-Supervised Classification with Graph Convolutional Networks” is a seminal work in the direction of graph convolutional neural networks (Kipf and Welling, 2016). Although Kipf is not a scholar of GNN in the field of drug discovery applications, his contributions have had a significant impact on the field’s development.

### 4.2 Research hot spots and trends

In bibliometric studies, paper citation analysis is an important tool to identify important papers, evaluate research progress, and predict research development frontiers. Highly cited papers are typically high-quality studies with strong innovation and significant impact. It is worth mentioning that the number of local citations is one of the important indicators to measure the influence of a paper, which reflects the importance of researchers in the same field. Table 7 displays the top 10 highly cited papers, each with more than 100 global citations and four with more than 50 local citations. Among these, the research paper “Modeling Polypharmacy Side Effects with Graph Convolutional Networks” published in Bioinformatics was cited 526 times, which is currently the most cited and was the only paper published in the field in 2018. In the paper, the authors propose a Decagon model for predicting side effects exhibited by drug combinations in clinical settings using graph convolutional neural networks. Altae-Tran H published another paper in 2017 that received nearly 400 citations, introducing a new architecture that, when combined with GCN, showed significant improvements in low-data predictive power in drug discovery. In third place was a paper published in 2020 about the application of graph attention mechanisms in drug discovery and development. However, with the increasing application of GNN technology in the drug discovery field, the method is likely to encounter additional questions and challenges. According to Table 7, Jiang’s research (Jiang et al., 2021) puts forward the point that an optimal predictive model should have a good balance between prediction accuracy and computational efficiency, but graph-based models have an overwhelmingly slower training speed than descriptor-based models, so graph-based models have more computational cost and less computational efficiency. Therefore, while the GNN is a rapidly emerging deep learning algorithm in the drug discovery field, there are still some issues that require further resolution and optimization.

Burst detection is an algorithm that can capture a sharp increase in the heat of a reference in a certain period of time and can be used as an effective method for identifying research hotspots and emerging trends over time. Figure 7 shows the top 25 references with the strongest citation bursts. The results of the study suggested that the first burst of reference citations in the field began in 2017 and continued until 2021. Steven Kearnes et al., 2016 published this paper on molecular graph convolution in the Journal of Computer-Aided Molecular Design in 2016. The paper argued that although molecular “fingerprinting” is the primary force in encoding structural information in current drug discovery, it has other shortcomings, such as the need to emphasize specific aspects of the molecular structure. They argued that molecular graph convolution can make better use of graph structural information, providing new methods and opportunities for improvements in virtual screening for drug discovery. This was also one of the earliest papers we discovered while collecting publication on applying GNN to drug discovery. From 2018 to 2023, Gilmer et al., 2017 paper has consistently garnered substantial citations. This paper describes a general framework for supervised learning on graphs called Message Passing Neural Networks (MPNNs). This framework abstracts commonalities among some of the most promising existing neural models designed for graph-structured data. The goal is to facilitate a better understanding of the relationships between these models and generate novel variations. Meanwhile, there are two references (Luo et al., 2017; Wen et al., 2017) citation outbreaks that are still ongoing, and both are related to DTI prediction, indicating that DTI is a hot research topic for the application of GNN in drugs.

According to Figure 8A, drug-target interaction prediction and drug-drug interaction prediction were the most frequent co-occurrence keywords for the range of drugs, which once again reflects that they are research hotspots in the application of GNN in drug discovery.

Most drugs achieve therapeutic effects through *in vivo* interactions with specific target molecules such as enzymes, nuclear receptors, G-protein-coupled receptors, and ion channels (Zhang et al., 2022a). Therefore, the identification of DTI is an important field of drug discovery. It is of great significance to develop effective computational methods for identifying DTI (Li et al., 2022a). For example, Zang’s team (Zhao et al., 2021) used drug networks and protein networks to generate drug-protein pairs (DPP) networks. In the DPP network, each node is a DPP, and the edges of the DPP network are inferred from the respective drug and protein networks. Here, DPP is a combination of any drug and any protein. If the drugs and proteins in a particular DPP can interact with each other, it is labeled as a true DPP and can be referred to as a DTI. The five unknown DTIs identified using the GCN-DTI model, supported by existing literature, demonstrate the reliability of the results and the effectiveness of GCN-DTI in recognizing real-world drug-target interactions. In recent studies, the application of multi-modal data features extraction and fusion techniques, such as node2vec and CNN, has been instrumental in enhancing the performance of DTI prediction. For example, Sajjad’s team (Dehghan et al., 2024) introduced a multimodal fusion CCL-DTI algorithm with contrastive loss. This method uses node2vec to extract features from protein-protein and drug-drug interaction networks, as well as 1D-convolutional neural networks to extract features from drug structures, protein sequences, and other data. Subsequently, a two-sided attention mechanism is utilized for the fusion of multi-modal features. Finally, a multi-layer perceptron is employed to predict the affinity value of DTI. Notably, during the MLP training process, the introduction and comparison of contrastive loss functions before evaluating the prediction loss function significantly enhanced the accuracy and reliability of the model. Similarly, Parvin’s team (Palhamkhani et al., 2023) proposed the DeepCompoundNet model, which shares similarities with the CCL-DTI algorithm. This model utilizes 1D-CNN and node2vec for feature extraction from proteins and compounds, as well as protein-protein and drug-drug interactions. Subsequently, based on the fused eigenvectors, the model determines the similarity between proteins and chemical vectors in the latent space and predicts interactions between them. These innovative GNN models, which are based on multi-modal data feature extraction and fusion, effectively capture and learn complex data patterns, resulting in significant improvements in DTI prediction performance.

The process of discovering new drugs is both expensive and time-consuming. Therefore, the exploration of novel target proteins that may be targeted is a crucial approach to repurposing drugs (Lu et al., 2017). It is well recognized that one drug may have an effect on several target proteins, and one target protein can be associated with multiple disorders. This forms the basis of drug repositioning. Therefore, drug repositioning is a form of drug-target interaction. GNN is also being widely used for drug repositioning. Lei’s team (Zhang et al., 2022b) proposed a new method based on Graph SAGE and clustering constraints (DRGCC) to investigate the potential therapeutic properties of drugs for drug repositioning. The team (Lei et al., 2022) also proposed a drug repositioning method for predicting drug-disease associations using a graph auto coder. These methods can be used to predict anti-COVID-19 drugs based on the existing drug and disease data.

The treatment of complex diseases by taking multiple medications is becoming increasingly popular, but it is equally important to circumvent the risk of drug-to-drug adverse reactions or unknown toxicity (Takeda et al., 2017). Therefore, the development of computational models for predicting drug-drug interactions (DDI) as preventable medical errors is also a research focus of GNN applied in the field of drugs, and good results have been achieved. Zitnik developed (Zitnik et al., 2018) Degacon, a method for predicting the side effects of drug pairs. First by constructing large multimodal maps of protein-protein interactions, drug-protein interactions, and drug-drug interactions, and then using modeling to process them in order to predict polypharmacy side effects. Zhang’s team (Feng et al., 2020) proposed a prediction model called DPDDI based on GCN and deep neural networks. GCN learns the low-dimensional feature representation of drugs by capturing the topological relationships of drugs in the DDI’s network. DPDDI can predict potential DDI without considering the chemical and biological properties of drugs. This solves the problem of high or unavailable acquisition costs for some drug properties. Although recent computational methods exhibit promising performance in DDI screening, their practical implementation faces two significant challenges: the necessity for comprehensive datasets for clinical utilization and the inference of DDI types for new drugs not encompassed in existing datasets. To address these obstacles, Yu’s team proposed (Feng et al., 2023) MM-GANN-DDI, a multimodal graph-agnostic neural network for predicting drug interaction events. The model was assessed using two datasets (DB-v1 and DB-v2) derived from the DrugBank database, which is a comprehensive resource that integrates biological and chemical information. It provides detailed data on drugs verified through experiments, making it a crucial source for studying DDI. Importantly, their model exhibits the potential to discover unobserved DDIs, demonstrating its practical application in clinical medication. Most of the above studies are done based on heterogeneous information networks, which can integrate different types of data in the form of graphs, providing rich information in drug discovery to help researchers obtain more accurate research results.

ADMET (absorption, distribution, metabolism, excretion, and toxicity) prediction was an important research direction emerging in the early stages (Liu et al., 2019), and the co-occurrence frequency of keywords such as molecular property prediction and molecular characterization was higher. Accurate prediction of molecular properties, such as physicochemical and bioactive properties, as well as ADMET properties, remains a fundamental challenge for molecular design, especially for drug design and discovery (Cai et al., 2022). Therefore, molecular property prediction is another research hotspot in this field.

As can be seen from Figure 8B, GCN was the central and developed theme. It can be preliminarily concluded that the GCN was the core algorithm of GNN in the field of drug application. According to Figures 8A,B, contrastive learning is a new method in this field. It is a method for self-supervised learning. Wang’s team (Wang et al., 2022b) proposed a MolCLR (Molecular Contrastive Learning of Representations via Graph Neural Networks) framework and showed that the contrastive learning framework significantly improved the performance of graph-neural-network encoders on various molecular property benchmarks, including both classification and regression tasks. And more sophisticated GNNs, which cannot utilize unlabeled data. Simple GNN models trained via MolCLR demonstrate significant improvements on all molecular benchmarks, benefited from pre-training on large unlabeled data, and improved the problem of insufficient data in molecular learning. The combination with transformer networks is also a recently popular combination, which can preserve the original information about the interactions between atoms in the chemical structure of a drug, overcoming the problem of a lack of learning of edge features by the graph convolutional neural network (Zhang et al., 2022c). In addition, improving the explainability of models is a common challenge for current machine learning models (Karimi et al., 2021; Verhaeghe et al., 2022). Existing methods to improve the interpretability of GNN are to introduce an attention mechanism (Karimi et al., 2021; Yang et al., 2022a), Yang’s team (Karimi et al., 2019) developed the Deep Affinity model by introducing an attention mechanism based on a unified RNN-CNN. It makes compound-protein affinity predictions easier to understand by measuring the importance of protein, compound, or pair-specific features. However, the graph attention mechanism only considers the neighborhood of a vertex (also known as masked attention), which cannot capture the global relationship between each molecule’s atoms. To this end, Chen’s team (Yang et al., 2022b) developed a novel visual explanation method, gradient-weighted affinity activation mapping (Grad-AAM), to analyze a deep learning model from the chemical perspective, which may help us gain chemical insights directly from data beyond human perception and improve the generalization and interpretation capability of drug target affinity (DTA) prediction modeling. Pablo’s team (Puentes et al., 2022) proposes a protein-ligand adversarial augmentation network (PLA-Net). PLA-Net is based on a gradient method to calculate antagonistic molecular amplification, thereby retaining biological consistency and essential class features in molecular graphs to improve the interpretability of target-ligand interactions (TLI) predictions.

Moreover, as shown in Figure 8C, GNN has been widely used in other areas closely related to drug discovery, such as drug response prediction (Liu et al., 2020; Nguyen et al., 2022), miRNA-disease association (Li et al., 2020; Li et al., 2022b), and protein-protein interaction (Schulte-Sasse et al., 2021; Yuan et al., 2022).

### 4.3 Summary and prospect

In light of the above, GNN has been widely applied in various fields of drug discovery, such as drug target interaction, drug-drug interaction, and ADMET prediction. These research results demonstrate that utilizing GNN for drug discovery can effectively reduce research and development costs and time, expedite the introduction of new drugs to the market, and facilitate faster entry of drugs into the clinical application stage. However, there are several challenges.

The first is interpretability. The GNN model is a black box with an opaque prediction process, which is especially important for drug discovery. For example, when the GNN’s prediction results recommend a certain molecule as a potential drug candidate, researchers need to understand the prediction process and the features that affect the prediction results in order to conduct further experimental verification. To improve the interpretability of GNN models, researchers optimized the GNN algorithm by introducing attention mechanisms and gradient methods. To a certain extent, this helps researchers understand the impact of data features on prediction results, but these methods only provide global interpretability and cannot provide detailed explanations for the decision-making logic for specific prediction aims.

The second is data availability and ethics. GNN models require large amounts of labeled data to support accurate predictions. Obtaining high-quality, comprehensive data is key to utilizing GNNs for drug discovery. The patient data and biometric information involved in this process need to strictly comply with ethical guidelines to protect patient privacy and data security. This issue has not garnered enough attention in current research. Furthermore, GNNs are known as black-box models, which complicates the interpretation of their predictions, which may affect the trust and acceptance of the models by clinical researchers and regulatory agencies. Therefore, GNN needs to formulate and follow scientific and reasonable specifications in drug discovery applications, which is crucial to ensuring data availability and regulatory compliance.

The third is the model’s computing resources. GNN models usually require extensive computing resources to train, especially when processing large-scale bioinformatics datasets. How to effectively utilize limited computing resources and improve model training efficiency is a problem that needs to be solved.

Therefore, developing novel GNN models that comply with clinical data ethics and regulatory frameworks, have high computational efficiency and predictive performance, lower computing resources, and better interpretability is a key technical issue to promote better drug discovery in the future.

## 5 Limitations

As far as we know, this is the first bibliometric thesis that maps and describes the application of GNN in the field of drug discovery. As far as we know, this is the first bibliometric thesis to chart and explain the application of GNN in drug discovery. In contrast to other narrative reviews, this thesis included multiple types of bibliometric software and tools for analysis and visualization, enhancing the analysis’s concreteness and intuitiveness while somewhat mitigating subjective biases. In addition, we conducted an analysis of the annual publication volume, publication countries, and publication institutions, providing a clear and complete overview of the research’s progress. Thus, researchers can accurately understand the field’s development. However, our study also has some limitations. Because of the limitations of the bibliometric software, our analysis was restricted to the texts available in the WoSCC database, potentially missing some publications not from WoSCC. Furthermore, we only studied English papers and may have missed non-English papers of exceptional quality. Future research may include more databases and languages, resulting in greater insights and findings in the field.

## 6 Conclusion

Our study investigated the current research status and hot spots of GNN in the field of drug discovery. Since 2021, the number of publications has increased dramatically. With reference to the annual publication volume trends, future research in this field will continue to increase and develop rapidly. So far, China and the United States have a significant advantage in terms of funding and the number of publications. They have also established good collaborations with other institutions and countries, as well as produced representative and high-quality papers. Other countries are also forming and growing small-scale research collaborations. More external collaborations between countries and institutions will encourage the creation of new research groups and the production of high-quality research. In addition, we also focused on the specific application of GNN in the prediction of DTI, DDI, and ADMET, discussed the new trend of GNN in the field of drug discovery, and summarized the practical significance and challenges of GNN in the field of drug discovery. In summary, our study utilized a bibliometric approach and provided the research status and hot spots of GNN in the field of drug discovery for researchers who will be engaged in this field, which has certain guiding values.

## Data Availability

The original contributions presented in the study are included in the article/supplementary material, further inquiries can be directed to the corresponding authors.

## Abbreviations

CNN, Convolutional Neural Networks; RNN, Recurrent Neural Networks; GNN, Graph Neural Networks; GCN, Graph Convolutional Networks; WoSCC, Web of Science Core Collection; TP, Total Publications; TC, Total Citations; IF, Impact Factor; GAT, Graph Attention Networks; DTI, Drug-Target Interactions; DPP, Drug-Protein Pairs; DDI, Drug-Drug Interactions; MolCLR, Molecular Contrastive Learning of Representations; ADMET, absorption, distribution, metabolism, excretion, and toxicity.
