# Supplementary material for: Knowledge mapping of graph neural networks for drug discovery: a bibliometric and visualized analysis
Source: Front Pharmacol. 2024 May 10;15:1393415. doi: 10.3389/fphar.2024.1393415 (PMC11116974; doi:10.3389/fphar.2024.1393415)
Supplement: Supplementary file 1 [file Table1.docx]

Supplementary Material

Knowledge Mapping of Graph Neural Networks for Drug Discovery: A Bibliometric and Visualized Analysis

# Supplementary Tables

**[Supplementary Table](javascript:;) 1.** Top 10 highly cited articles in China

| Rank | Title | Source | Pub. year | Document Type | Citations | IF (2023) |
| --- | --- | --- | --- | --- | --- | --- |
| 1 | Pushing the boundaries of molecular representation for drug discovery with the graph attention mechanism | Journal of Medicinal Chemistry | 2020 | Article | 274 | 7.3 |
| 2 | [Deep learning in bioinformatics: Introduction, application, and perspective in the big data era](https://webofscience.clarivate.cn/wos/woscc/full-record/WOS:000483008800002) | Methods | 2019 | Article | 168 | 4.8 |
| 3 | Could graph neural networks learn better molecular representation for drug discovery? A comparison study of descriptor-based and graph-based models | Journal of Cheminformatics | 2021 | Article | 167 | 8.6 |
| 4 | Predicting drug-disease associations through layer attention graph convolutional network | Briefings in Bioinformatics | 2021 | Article | 136 | 9.5 |
| 5 | Identifying drug-target interactions based on graph convolutional network and deep neural network | Briefings in Bioinformatics | 2021 | Article | 122 | 9.5 |
| 6 | Drug-target affinity prediction using graph neural network and contact maps | Rsc Advances | 2020 | Article | 105 | 3.9 |
| 7 | [Deep Learning-Based Prediction of Drug-Induced Cardiotoxicity](https://webofscience.clarivate.cn/wos/woscc/full-record/WOS:000462943700013) | Journal of Chemical information and Modeling | 2019 | Article | 96 | 5.6 |
| 8 | [Graph Neural Networks and Their Current Applications in Bioinformatics](https://webofscience.clarivate.cn/wos/woscc/full-record/WOS:000684667800001) | Frontiers in Genetics | 2021 | Article | 68 | 3.7 |
| 9 | DPDDI: a deep predictor for drug-drug interactions | Bmc Bioinformatics | 2020 | Article | 67 | 3 |
| 10 | A self-attention based message passing neural network for predicting molecular lipophilicity and aqueous solubility | Journal of Cheminformatics | 2020 | Article | 67 | 8.6 |

**[Supplementary Table](javascript:;) 2.** Funding Organizations

| Rank | Funding Agency | Documents | Countries |
| --- | --- | --- | --- |
| 1 | National Natural Science Foundation of China NSFC | 284 | China |
| 2 | National Key Research and Development Program of China | 43 | China |
| 3 | National Institutes Of Health Nih USA | 33 | USA |
| 4 | United States Department of Health Human Services | 33 | USA |
| 5 | Fundamental Research Funds for The Central Universities | 29 | China |
| 6 | National Science Foundation NSF | 29 | USA |
| 7 | National Key R&D Program of China | 25 | China |
| 8 | National Research Foundation of Korea | 23 | Korea |
| 9 | Natural Science Foundation Of Zhejiang Province | 13 | China |
| 10 | European Union Eu | 12 | European Union |
| 10 | Ministry of Science Ict Future Planning Republic of Korea | 12 | Korea |
